# Adherence to Time-Restricted Feeding and Impact on Abdominal Obesity in Primary Care Patients: Results of a Pilot Study in a Pre–Post Design

**DOI:** 10.3390/nu11122854

**Published:** 2019-11-21

**Authors:** Dorothea Kesztyüs, Petra Cermak, Markus Gulich, Tibor Kesztyüs

**Affiliations:** 1 Institute of General Practice, Ulm University, Helmholtzstr. 20, 89081 Ulm, Germany; petra-1.cermak@uni-ulm.de (P.C.); markus.gulich@uni-ulm.de (M.G.); 2 Institute of Medical Informatics, Georg-August University, Von-Siebold-Str. 3, 37075 Göttingen, Germany; tibor.kesztyues@med.uni-goettingen.de

**Keywords:** fasting, noncommunicable diseases, lifestyle, obesity, abdominal, general practitioners, pilot project

## Abstract

The epidemic of lifestyle-dependent diseases and the failure of previous interventions to combat the main causes demand an alternative approach. Abdominal obesity is associated with most of these diseases and is a good target for therapeutic and preventive measures. Time-restricted feeding (TRF) offers a low-threshold, easy-to-implement lifestyle-modification concept with promising results from animal testing. Here, we describe a pilot study of TRF with abdominally obese participants (waist-to-height ratio, WHtR ≥0.5) in a general practitioner’s office. Participants (*n* = 40, aged 49.1 ± 12.4, 31 females) were asked to restrict their daily eating time to 8–9 hours in order to prolong their overnight fasting period to 15–16 hours. Questionnaires, anthropometrics, and blood samples were used at baseline and at follow-up. After three months of TRF, participants had reached the fasting target, on average, on 85.5 ± 15.2% of all days recorded. Waist circumference (WC) was reduced by −5.3 ± 3.1cm (*p* < 0.001), and three participants reached a WHtR <0.5. HbA1c was diminished by −1.4 ± 3.5 mmol/mol (*p* = 0.003). TRF may be an easily understandable and readily adoptable lifestyle change with the potential to reduce abdominal obesity and lower the risk for cardiometabolic diseases. Further well-designed studies are necessary to investigate the applicability and usefulness of TRF for public health.

## 1. Introduction

Noncommunicable diseases (NCDs) are mainly lifestyle dependent and pose an enormous threat to modern societies. The WHO numbers of global deaths due to NCDs are markedly high, reaching more than 90% in industrialized countries [1,2]. A significant proportion of those NCD deaths are premature, and although life expectancy in most countries is rising, the US has recently seen a decline [3]. Most of the NCDs are strongly linked to obesity, more precisely to abdominal obesity [4,5,6,7], but despite countless efforts to curb the continuously increasing prevalence of it, successful therapeutic interventions, especially for abdominal obesity, are rare [8]. However, there is evidence for various diet programs that support weight loss in general, as long as the diet is maintained [9], and, particularly, bariatric surgery is successful, but reserved for only a small part of those affected [10].

In recent years, intermittent fasting has attracted more attention from scientists and sparked interest among the general population. Many studies have already been conducted on intermittent fasting and have shown that this type of fasting is safe and effective [11]. Time-restricted feeding (TRF) as a special form of intermittent fasting was extensively tested in animal experiments [12,13]. Only a little data from small and partially uncontrolled studies are available for humans, but the results of these studies are also promising, especially with regard to metabolic factors. The relevant studies are described below. 

Restricting daily eating hours from initially >14 to 10–11 hours for 16 weeks reduced body weight on average by 3.27 kg (95% CI: 0.91–5.62 kg) and improved sleep in 8 overweight individuals [14]. Thirteen or more hours fasting per night was associated with a lower risk of breast cancer recurrence in 2413 women in an epidemiological study [15]. Eight to nine hours TRF in a pilot study resulted in favorable effects on energy intake, adiposity, and metabolic markers after 10 weeks in seven participants, compared to the controls [16]. Eight hours of TRF reduced body weight, energy intake, and blood pressure in 29 obese participants in a 12 week pilot study with a historical control group [17]. Fasting for 16 hours per day for eight weeks leads to decreases in testosterone, insulin-like growth factor 1, and triiodothyronine, but showed no negative impact on muscle mass and strength in 17 resistance-trained males compared to a control group [18]. Early TRF for six hours improved insulin sensitivity, blood pressure, and oxidative stress, even without weight loss, in four prediabetic men in a controlled feeding study of five weeks [19]. Following a six-hour TRF (eating phase starting early in the morning) schedule for four days suggested an altered expression of circadian clock genes of several genes associated with antiaging and changed lipid metabolism in 11 overweight adults in comparison to a control schedule [20]. Four hours TRF on four days and unrestricted eating on three days (with resistance training) reduced energy intake, but did not adversely affect muscular improvements in 10 young men in a randomized trial [21]. Finally, we conducted an investigation among employees of the university and the clinic in Ulm on the compatibility of TRF with a professional activity. We were able to show that the majority of the participants had little or no problems with the implementation of TRF (unpublished data).

We hypothesize that TRF may be suitable as a low-threshold intervention that may be delivered in a primary care setting. Given the positive aspects of TRF from the above studies, we wanted to investigate the feasibility of conducting TRF with patients in a general practitioner’s (GP) office since, to our knowledge, TRF had not yet been tested there. Because TRF is an increasingly popular dietary intervention which may help people to change their lifestyles, a GP’s office seems like a reasonable place to initiate a TRF intervention and to determine whether there were any accompanying health benefits. Hence, as a measure of feasibility and adherence, the percentage of days with reaching the fasting target in the total of the recorded days is reported, together with other information on feasibility and acceptance by patients in general practice.

We focused on otherwise healthy patients with components of the metabolic syndrome (METS). According to the practice-oriented definition of the International Diabetes Federation (IDF), abdominal obesity is a required criterion in the presence of two out of four other factors (raised triglycerides, reduced HDL cholesterol, raised blood pressure, and raised fasting plasma glucose) [22]. Other definitions of the METS also include measures of abdominal obesity. In this respect, the waist circumference (WC) is the most commonly used measure and is a modifiable factor, along with body weight or BMI, sensitive to lifestyle changes [8]. Our hypothesis was that TRF would lead to a reduction in abdominal obesity; therefore; we examined changes in waist circumference (WC) and waist-to-height ratio (WHtR) as proxies for intra-abdominal fat [23]. 

## 2. Materials and Methods

Due to the pilot character of this study, it was planned in a pre–post design without a control group. The study took place in a medium-sized general practitioner’s practice whose owner was part of the study team. The intervention period started in March 2019, and data acquisition was completed in June 2019. The primary outcome, as stated in the protocol, was the proportion of days in which participants reached the fasting objective (≥15 hours) out of the total number of days recorded by each participant. Pre–post differences in anthropometrics and METS related blood parameters, among others, were secondary outcomes.

Written informed consent was available for all participants included in the study. The ethics commission of the State Medical Chamber of Baden-Württemberg approved the study (March 2019, Application No. B-F-2019-022). The study was registered on the German Clinical Trials Register (DRKS) under the DRKS-ID: DRKS00015057.

### 2.1. Recruiting

Patients were invited during a consultation by the doctor to participate in the study or informed about the study through flyers in the waiting room of the general practitioner’s office. Inclusion criteria were one or more components of the metabolic syndrome, including diabetes type 2 not requiring insulin. Exclusion criteria were diabetes type 1, hyperthyroidism, pregnancy, any eating disorder, or any condition where fasting was contraindicated [24]. No patients were excluded who wanted to participate. Two patients who were invited by the doctor declined, one because TRF did not fit into the families’ time schedule of eating, and one eligible participant who expressed concern that he would be unable to adhere to the intervention. Finally, 40 participants were recruited. 

### 2.2. Intervention

Participants were asked to limit the daily period of their food intake to 8–9 hours and to extend their nightly fasting period to 15–16 hours as a result. The duration of the intervention was 3 months. The participants were thoroughly informed about the intervention in a 30-minute personal consultation with the GP. For each participant, the best period of food intake and fasting was determined according to individual preference. Participants were not asked to change the composition of their usual diet. They received a diary to document the daily beginning, and end of, food intake. After about 2–3 weeks, participants were contacted by the doctor’s office to discuss any problems that might have arisen or to answer any questions. During these structured telephone conversations, participants were asked how they felt about TRF, how they were coping, whether there were any difficulties, and whether they had any side effects. These conversations of at least five minutes were conducted by trained medical assistants and were documented. Throughout the entire duration of the trial, the participants were able to contact or visit the office and get help and support.

### 2.3. Data Assessment

Baseline consultations started in parallel with recruitment in March 2019 and continued until 40 participants were enrolled. Participants were informed in detail about the content and aims of the study, received an information brochure, and completed a short questionnaire in order to give some information on their personal lifestyle and health behavior. They were asked about their eating, drinking, snacking, smoking, physical activity, and screen-media behavior. They reported medication and supplement use. Consumption of sweetened beverages, fruits and vegetables, processed food, sweet snacks, and salty snacks was assessed on a 6-point Likert-scale, ranging from never to several times a day. The same scale was used for physical activity, defining one unit as 30 minutes of moderate to vigorous activity. Screen-media use was assessed on a 7-point Likert-scale, from never to more than 6 hours per weekday or weekend day. Questions were based on questionnaires from previous studies or surveys [25,26].

The GP was trained by an author with a certificate from the International Society for the Advancement of Kinanthropometry (ISAK) and performed the anthropometric measurements according to a standardized protocol based on ISAK [27]. Body weight was measured in light clothing to the nearest 0.1 kg, with a calibrated and balanced portable digital scale (model 862, seca®, Hamburg, Germany). Height was determined to the nearest 0.1 cm, with a portable height-measuring instrument (Stadiometer, seca®, Hamburg, Germany). WC was taken to the nearest 0.1 cm at umbilical level with a flexible metal tape (Lufkin Industries Inc., Lufkin, Texas, USA). The WHtR was calculated as the quotient of waist and height in cm, and, by applying a threshold of 0.5, as recommended [28], abdominal obesity was determined. The body mass index (BMI) was determined (body weight/height in meters^2^), and normal weight (<25), overweight (≥25), and obesity (≥30) were defined. 

Both the baseline and the follow-up examination took place in the morning in a fasting state of the participants. A blood sample was drawn to examine inflammatory and metabolic parameters, namely cholesterol, low-density lipoprotein (LDL), high-density lipoprotein (HDL), triglycerides, glycosylated hemoglobin (HbA1c), and high-sensitivity C-reactive protein (hsCRP). Serum levels of cholesterol, HDL, LDL, and triglycerides were measured spectrophotometrically by a direct enzymatic colorimetric method, and hsCRP was determined on a latex-enhanced turbidimetric immunoassay method. HbA1c was measured by high-performance liquid chromatography (HPLC) on whole blood samples. All analyses were provided by a certified contract laboratory.

After three months, the same data collection procedure was repeated during follow-up, but the questionnaire was more comprehensive and included questions on the feasibility, effects, and side effects, and about experience with and attitude toward TRF. Furthermore, participants were asked to rate the subjectively perceived effects of TRF on their health on a 5-point Likert-scale, ranging from very positive to very negative. 

As far as applicable, all values are reported in Système international d’unités (SI) units.

### 2.4. Statistical Analysis

Baseline characteristics were depicted descriptively for the whole group, and separately for men and women. Differences between groups were tested according to scale level and distribution, applying Fisher’s exact test for categorical data and Mann–Whitney U test, t-test, or Welch’s t-test (considering variance heterogeneity) for continuous data.

Pre–post differences were calculated and are displayed as the respective Δ of the corresponding variable. In order to measure the significance of the differences between pre and post, the Wilcoxon signed-rank test for related samples was used.

In the diary data, the individual mean values for time of first meal, time of last meal, eating phase, and fasting phase were calculated for each participant and, based on this, the overall mean and standard deviation. The percentage of days with achievement of the fasting target was also calculated per participant, and then reported as a group mean with standard deviation.

Bivariate correlations between continuous variables were examined with the Pearson correlation coefficient.

The analysis of pre–post data followed an intention-to-treat approach, the missing values of the two dropouts for the final examination were replaced according to the principle of last information carried forward in this case baseline data. 

The significance level was set at α = 0.5 for two-sided tests. All statistical analyses were carried out by using the statistical software packages IBM SPSS Statistics for Windows, Version 25.0. (IBM Corp., Armonk, NY, USA).

## 3. Results

Of the 40 participants recruited, one discontinued the intervention due to personal overload and one other for unknown reasons. At baseline, one hsCRP value was excluded from analysis because it was >100 mg/L due to an acute infection shortly before starting the intervention, but at the time of the blood collection, the antibiotic treatment was already completed, and the participant was free from symptoms. 

### 3.1. Participant Characteristics

A considerable proportion of participants (63%, *n* = 35) reported daily medication. The most common medication was antihypertensive and hypothyroidism drugs. There was only one participant with a prescription for a cholesterol-lowering agent (statin). 

The baseline characteristics of all participants are depicted in Table 1.

Differences between male and female participants occurred in weight, where women weighed significantly less than men; in HDL, where women had significantly higher values than men; and in triglycerides, where women had significantly lower values than men. All included participants were abdominally obese (WHtR ≥ 0.5).

### 3.2. Primary Outcome and Diaries

**Figure 1:**
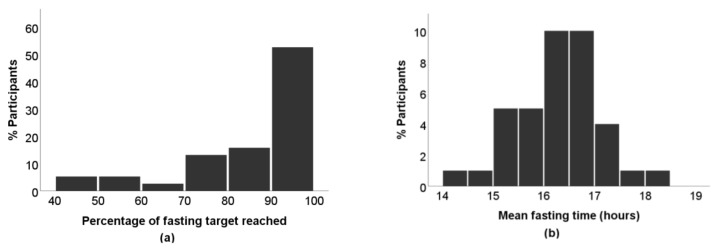
(**a**) Percentage of days where the fasting target was reached of all recorded days per participant, *x* axis subdivided into categories of 10% each; (**b**) mean fasting hours of all recorded days per participant, *x* axis subdivided into categories of 0.5 hours each.

Participants reached the fasting target of 15–16 hours on average in 86 ± 15% of all recorded days, as depicted in Figure 1a, ranging from 40% to 100%. More information from diaries concerning the timing of the first and last meal, daily eating, and fasting hours are presented in Table 2. 

There were no statistically significant differences between men and women. A graphical representation of the average fasting time is shown in Figure 1b.

### 3.3. Secondary Outcomes

Participants experienced moderate weight loss (−1.7 ± 2.5 kg) and corresponding changes in BMI (−0.6 ± 0.9) after three months of TRF, but lost −5.3 ± 3.1 cm WC, leading to a reduction in WHtR of −0.03 ± 0.02, and, subsequently, three (7.5%) participants were no longer classified as abdominally obese. Four overweight and one obese participant (12.5%) lost enough weight to fall under the BMI threshold of 25 for normal weight. Weight loss was inversely correlated with the percentage of fasting target reached (*r* = 0.435, *n* = 38, *p* = 0.006), but there was no significant correlation found for WC (*r* = −0.046, *n* = 38, *p* = 0.783). HbA1c was reduced by −1.4 ± 3.5 mmol/mol, which correlated with weight loss (*r* = 0.412, *n* = 38, *p* = 0.008) and changes in BMI (*r* = 0.367, *n* = 38, *p* = 0.020), but not with changes in WC, fasting or eating hours, or fasting target reached. At baseline, five participants had HbA1c values ≥42 mmol/mol, and, after three months of TRF, there were three participants left with elevated values, but these were also reduced on average by 11 mmol/mol. A comprehensive overview is given in Table 3.

### 3.4. Feelings of Hunger, Side Effects, and Participants’ Assessment

Feeling hungry every day was reported by three (8%), several days per week by 10 (25%), once a week or less by 22 (58%), and never by three (8%) participants. Cravings, dizziness, nausea, or other side effects occurred in seven (18%), five (13%), two (5%), and one (3%) participant(s), respectively. Nine participants (23%) experienced these side effects at the beginning of the intervention, less than once a week, or once a week, while three (8%) participants complained several times per week, or on a daily basis. Five (13%) participants reported no improvement of these side effects, whereas eight (20%) participants felt an improvement in the course of the study. Twenty-five (63%) participants stated that they had not noticed any of these side effects. There were no significant differences in the achievement of the fasting target between the participants with and without side effects.

During the telephone contacts 2–3 weeks after fasting initiation, participants reported how they coped with fasting, whether they noticed changes, how they felt about their health, and which problems or questions arose. Initial difficulties were reported, including hunger in five participants and difficulties with shift work; one participant felt impaired during sports; one participant complained about indigestion; some participants reported it took them a few days until a rhythm was established; and some felt fitter, slept better, and felt more comfortable. 

There were no significant pre–post differences in the consumption of sweetened beverages, fruits and vegetables, or processed foods. Sweet snacks and salty snacks were both significantly reduced (*p* < 0.001). There were no changes in physical activity or screen-media consumption.

Twenty-three (40%) participants found it easy or very easy to adhere to TRF rules, 11 (28%) found it neither easy nor difficult, but four (10%) participants found it difficult or very difficult. Twenty-five (55%) participants stated that they did not deviate from the TRF rules at all or deviated from them less than once a week; eight (21%) deviated once a week; four (10%) deviated several times a week; and one deviated daily. Twenty-five (63%) participants were able to combine TRF very well or well with their daily work routine, six (15%) badly or very badly, six (15%) were in between, and one was not employed. 

Most participants (*n* = 29, 76%) felt positive or very positive, eight (21%) neither positive nor negative, and one very negative about TRF’s effects on their health. Fifteen (37%) participants said they wanted to continue, 20 (53%) were undecided, and three (8%) did not want to continue TRF. Thirty-one (78%) participants said they would recommend TRF, six (15%) were undecided, and one would not recommend TRF to others.

## 4. Discussion

TRF was predominantly well accepted by participants. They reached the primary outcome, days with fasting target achieved on all days recorded, on average in 86% of cases. This was more than expected, since, in a previous pilot study with 63 employees of Ulm University and hospital, the fasting target was achieved on 72.2 ± 18.9% of recorded days (unpublished data). This difference may have been partly due to closer supervision in the GP’s office. Therefore, the adherence of the participants to the TRF protocol in the GP’s office was excellent, and this is also reflected in the other results. However, all but three participants reported hunger, and 13 (21%) experienced side effects, such as cravings, dizziness, and nausea, but eight of them reported improvements over time. Considering this, having six (15%) participants who reached the fasting target on less than 75% of all days recorded seems fairly low. Apart from side effects, some participants had difficulties bringing their TRF schema into accordance with their working schedule, which has of course led to some deviations from the proposed eating/fasting plan. 

The mean reduction in WC of −5.3 ± 3.1 cm was considerable, especially in comparison with an overall reduction of −2.7 cm as the leading result of a meta-analysis of therapeutic options for abdominal obesity in adults [8], although one has to keep in mind the higher quality of the randomized controlled trials in the systematic review compared to this study in an uncontrolled pre–post design. The participants in the aforementioned pilot study at Ulm University lost only −1.7 ± 3.22 cm WC, but the initial value there was also significantly lower (89 vs. 107 cm) (unpublished data). 

The reduction of HbA1c was also small, but statistically significant, with −1.4 mmol/mol between baseline and follow-up, and, with respect to the initial value of 37.5 mmol/mol, achieved a decrease of −3.8%. From five participants with initially elevated HbA1c levels, 2 managed to fall below the threshold of 42 mmol/mol at follow-up. Interestingly, these positive results were achieved without major dietary changes, except for a reduction in snacking behavior. Hence, an improvement of diet can possibly further enhance the results. Taken together, these are promising results that need to be confirmed in randomized, controlled trials.

Contrary to investigations in animals, no significant changes in the lipid profile were observed in this study. This fact may be due to an only moderate weight loss of 1.7 ± 2.5 kg. Other researchers did not investigate blood lipids [14,21], or did not detect any changes after eight weeks of TRF [18]. Another study, just like ours, found no differences in HDL, LDL, and triglycerides after 12 weeks of TRF in obese participants [17]. Sutton et al. reported increased morning values of triglycerides and total cholesterol, but no changes in LDL and HDL, in their study of early TRF [19]. Jamshed et al., similar to Sutton et al., found increases in HDL and LDL cholesterol values in the morning [20]. Finally, one study observed nonsignificant downward trends in LDL after 10 weeks of TRF in 16 healthy participants [16]. The impact of TRF and especially the timing of food intake on blood lipids have to be investigated more intensively in future studies. 

Various mechanisms of TRF are discussed that may lead to health benefits. A synergistic interaction between chronobiological circadian rhythms [29], intestinal bacteria (“microbiome”) [30], the immune system [31] and autophagy is discussed [20,32]. TRF may be considered as a natural means to strengthen the homeostasis of health, as it brings people closer to the millennia-old rhythm of eating and fasting. Research on shift work has shown that shifts have a negative effect on health [33,34]. It is particularly noteworthy that earlier ingestion of food may increase the positive effect of TRF because it is better tuned to the intrinsic metabolic circadian rhythmicity [19,20,35,36,37].

### Strengths and Limitations

The dropout of two participants can be considered as fairly low. Nonetheless, where necessary, their baseline data were carried forward to adhere to the intention-to-treat analysis. A dropout rate of 5% is acceptable, as case number calculations are often made at much higher dropout rates [38]. Other kinds of missing values from examinations or questionnaires did not appear. The greatest strength of this pilot study lies in the very good adherence of the participants, which may largely be due to the stable attachment of the participants to the staff and the GP’s office. All in all, the study provides many useful findings for the conception of further investigations of TRF in primary care.

The most obvious limitation is the study design without a control group. This fact can be attributed to the pilot character of the study, which was intended to provide data for the design of further studies and had to get by without funding. Furthermore, for the primary outcome adherence to the TRF protocol, a control group was not necessary. A further limitation is the measurement of the secondary outcome WC, which was, not in full accordance with the ISAK protocol [27], not constantly taken twice. Similar to our first pilot study, men were underrepresented, a phenomenon that is also discussed in the literature, where middle-aged and older male participants are underrepresented in research of health promotion and prevention [39], while women are partly missing in clinical studies [40].

Several sources of bias may have occurred. Sex bias is already depicted above; selection bias is, on the one hand, due to the study design, and, on the other hand, due to the method of recruitment, affecting both the doctor’s selection and the self-selection of participants. Performance and detection bias occurred because no blinding was applied. Attrition bias because of incomplete outcome data was low, since only two participants failed to complete the intervention and all other data were available. Social desirability bias may have arisen in the questionnaires and diaries, and, hence, self-reported hours of eating and fasting may not have been recorded accurately. 

Taken together, these results are mainly exploratory and should be interpreted with caution. 

## 5. Conclusions

Despite hunger and side effects, participants adhered quite well and only a few did not reach their fasting target on at least 75% of the days reported. TRF may help to reduce abdominal obesity and hence prevent cardio-metabolic diseases. TRF may be suitable for application in primary care for interested patients with appropriate counseling and patient management. Further well-designed studies are necessary to investigate the applicability, usefulness, and cost-effectiveness of TRF for public health. 

## Figures and Tables

**Table 1:** Baseline characteristics of participants in the TRF-GP pilot study 2019.

	Female (*n* = 31)	Male (*n* = 9)	Total (*n* = 40)
Age, years M (SD)	49.2 (11.3)	48.6 (16.2)	49.1 (12.4)
Weight, kg M (SD)	**84.2 (18.3) ^1^**	104.6 (23.6)	88.8 (21.1)
Waist circumference, cm M (SD)	104.7 (11.5)	114.3 (17.0)	106.9 (13.3)
BMI, kg/m^2^ M (SD)	31.1 (6.0)	31.9 (5.7)	31.3 (5.9)
WHtR, M (SD)	0.64 (0.07)	0.63 (0.09)	0.64 (0.07)
Normal weight, *n* (%)	4 (12.9)	1 (11.1)	5 (12.5)
Overweight, *n* (%)	10 (32.3)	3 (33.3)	13 (32.5)
Obese, *n* (%)	17 (54.8)	5 (55.6)	22 (55.0)
Abdominal obese, *n* (%)	31 (100)	9 (100)	40 (100)
HbA1c, mmol/mol	36.6 (5.6)	40.4 (13.4)	37.5 (8.0)
LDL, mmol/L M (SD)	3.4 (1.1)	2.85 (1.1)	3.3 (1.1)
HDL, mmol/L M (SD)	**1.5 (0.4) ^2^**	1.0 (0.3)	1.4 (0.4)
TCHOL, mmol/L M (SD)	6.0 (1.2)	4.7 (1.1)	5.4 (1.2)
Triglycerides, mmol/L M (SD)	**1.2 (0.7) ^3^**	2.1 (0.9)	1.4 (0.8)
hsCRP, mg/L M (SD) *	2.69 (2,36)	1.47 (1.23)	2.41 (2.20)
Daily eating time, h M (SD)	12.40 (2.15)	11.13 (2.81)	12.23 (2.27)

NOTE: TRF = time-restricted feeding; GP = general practitioner; M = mean; SD = standard deviation; kg = kilogram; cm = centimeter; h = hours; BMI = body mass index; WHtR = waist-to-height ratio; HbA1c = glycosylated hemoglobin; LDL = low density lipoprotein; HDL = high density lipoprotein; TCHOL = total cholesterol, hsCRP = high-sensitivity C-reactive protein; * 1 value excluded; significant values are bold; ^1^ *p* = 0.037, ^2^ *p* < 0.001, ^3^ *p* = 0.001.

**Table 2:** Diary of participants in the TRF-GP pilot study 2019.

	Female (*n* = 30)	Male (*n* = 8)	Total (*n* = 38)
Time of first meal, M (SD)	10.57 (1.72)	9.58 (1.92)	10.36 (1.78)
Time of last meal, M (SD)	18.24 (1.70)	17.35 (1.31)	18.05 (1.65)
Eating phase, h M (SD)	7.74 (0.71)	7.71 (1.23)	7.73 (0.82)
Fasting phase, h M (SD)	16.24 (0.69)	16.30 (1.23)	16.25 (0.81)
Fasting target reached, % M (SD)	85.9 (14.6)	83.8 (18.1)	85.5 (15.2)

NOTE: TRF = time-restricted feeding; GP= general practitioner; M = mean; SD = standard deviation; h = hours.

**Table 3:** Follow-up results of participants in the TRF-GP pilot study 2019.

	Female (*n* = 31)	Male (*n* = 9)	Total (*n* = 40)
Weight, kg M (SD)	**82.3 (18.5) ^1^**	103.7 (24.7)	87.1 (21.7)
Waist circumference, cm M (SD)	99.4 (12.7)	109.2 (17.4)	101.6 (14.2)
BMI, kg/m^2^ M (SD)	30.4 (6.1)	31.5 (5.9)	30.7 (6.0)
WHtR, M (SD)	0.61 (0.07)	0.60 (0.09)	0.61 (0.08)
Normal weight, *n* (%)	9 (29.0)	1 (11.1)	10 (25.0)
Overweight, *n* (%)	6 (19.4)	3 (33.3)	9 (22.5)
Obesity, *n* (%)	16 (51.6)	5 (55.6)	21 (52.5)
Abdominal obesity, *n* (%)	29 (93.5)	8 (88.9)	37 (92.5)
HbA1c, mmol/mol	35.2 (4.1)	38.7 (7.8)	36.0 (5.3)
LDL, mmol/L M (SD)	3.6 (1.1)	3.0 (1.2)	3.5 (1.2)
HDL, mmol/l M (SD)	**1.5 (0.3) ^2^**	1.0 (0.3)	1.4 (0.4)
TCHOL, mmol/L M (SD)	5.6 (1.3)	4.8 (1.2)	5.4 (1.3)
Triglycerides, mmol/L M (SD)	**1.2 (0.8) ^3^**	2.0 (1.4)	1.4 (1.0)
hsCRP, nmol/L M (SD) *	2.63 (2.43)	2.28 (2.74)	2.55 (2.47)
Δ Weight, kg M (SD)	−1.93 (1.93)	−0.92 (4.05)	**−1.71 (2.53) ^a1^**
Δ Waist circumference, cm M (SD)	−5.29 (3.15)	−5.12 (3.12)	**−5.** **26 (3.10) ^a1^**
Δ BMI, M (SD)	−1.01 (0.75)	−0.32 (1.27)	**−** **0.64 (0.89) ^a1^**
Δ WHtR, M (SD)	−0.03 (0.02)	−0.03 (0.02)	**−** **0.03 (0.02) ^a1^**
Δ HbA1c, mmol/mol M (SD)	−1.4 (2.4)	−1.7 (6.0)	**−1.4 (3.5) ^a2^**
Δ LDL, mmol/L M (SD)	0.2 (0.5)	0.2 (0.4)	0.2 (0.5)
Δ HDL, mmol/L M (SD)	0.0 (0.1)	0.1 (0.1)	0.0 (0.1)
Δ TCHOL, mmol/L M (SD)	0.0 (0.4)	0.1 (0.3)	0.0 (0.4)
Δ Triglycerides, mmol/L M (SD)	0.0 (0.4)	−0.1 (1.3)	0.0 (0.7)
Δ hsCRP, nmol/L M (SD) *	−0.10 (1.86)	0.82 (3.05)	0.11 (2.18)

NOTE: TRF = time-restricted feeding; GP = general practitioner; M = mean; SD = standard deviation; kg = kilogram; cm = centimeter; BMI = body mass index; WHtR = waist-to-height ratio; HbA1c = glycosylated hemoglobin; LDL = low density lipoprotein; HDL = high density lipoprotein; TCHOL = total cholesterol, hsCRP = high-sensitivity C-reactive protein; * 1 value excluded; significant values are bold; ^1^ *p* = 0.024, ^2^ *p* = 0.001, ^3^ *p* = 0.018; Δ = differences between baseline and follow-up; ^a^ significance of differences between baseline and follow-up, ^a1^ *p* < 0.001, ^a2^ *p* = 0.003.
